# A New Online Dynamic Nomogram: Construction and Validation of an Assistant Decision-Making Model for Laryngeal Squamous Cell Carcinoma

**DOI:** 10.3389/fonc.2022.829761

**Published:** 2022-05-26

**Authors:** Yuchen Liu, Yanxun Han, Bangjie Chen, Jian Zhang, Siyue Yin, Dapeng Li, Yu Wu, Yuan Jiang, Xinyi Wang, Jianpeng Wang, Ziyue Fu, Hailong Shen, Zhao Ding, Kun Yao, Ye Tao, Jing Wu, Yehai Liu

**Affiliations:** ^1^Department of Otolaryngology, Head and Neck Surgery, The First Affiliated Hospital of Anhui Medical University, Hefei, China; ^2^Anhui Medical University, Hefei, China; ^3^Department of Oncology, The First Affiliated Hospital of Anhui Medical University, Hefei, China; ^4^Department of Otolaryngology, Head and Neck Surgery, The Fuyang Hospital Affiliated to Anhui Medical University, Fuyang, China

**Keywords:** laryngeal squamous cell carcinoma, dynamic nomogram, diagnosis, risk factors, LASSO regression

## Abstract

**Background:**

Laryngeal squamous cell carcinoma (LSCC) is the most common type of head and neck squamous cell carcinoma. However, there are currently no reliable biomarkers for the diagnosis and prognosis of LSCC. Thus, this study aimed to identify the independent risk factors and develop and validate a new dynamic web-based nomogram that can predict auxiliary laryngeal carcinogenesis.

**Methods:**

Data on the medical history of 221 patients who were recently diagnosed with LSCC and 359 who were recently diagnosed with benign laryngeal lesions (BLLs) at the First Affiliated Hospital of Anhui Medical University were retrospectively reviewed. Using the bootstrap method, 580 patients were divided in a 7:3 ratio into a training cohort (LSCC, 158 patients; BLL, 250 patients) and an internal validation cohort (LSCC, 63 patients; BLL, 109 patients). In addition, a retrospective analysis of 31 patients with LSCC and 54 patients with BLL from Fuyang Hospital affiliated with Anhui Medical University was performed as an external validation cohort. In the training cohort, the relevant indices were initially screened using univariate analysis. Then, least absolute shrinkage and selection operator logistic analysis was used to evaluate the significant potential independent risk factors (P<0.05); a dynamic online diagnostic nomogram, whose discrimination was evaluated using the area under the ROC curve (AUC), was constructed, while the consistency was evaluated using calibration plots. Its clinical application was evaluated by performing a decision curve analysis (DCA) and validated by internal validation of the training set and external validation of the validation set.

**Results:**

Five independent risk factors, sex (odds ratio [OR]: 6.779, P<0.001), age (OR: 9.257, P<0.001), smoking (OR: 2.321, P=0.005), red blood cell width distribution (OR: 2.698, P=0.001), albumin (OR: 0.487, P=0.012), were screened from the results of the multivariate logistic analysis of the training cohort and included in the LSCC diagnostic nomogram. The nomogram predicted LSCC with AUC values of 0.894 in the training cohort, 0.907 in the internal testing cohort, and 0.966 in the external validation cohort. The calibration curve also proved that the nomogram predicted outcomes were close to the ideal curve, the predicted outcomes were consistent with the real outcomes, and the DCA curve showed that all patients could benefit. This finding was also confirmed in the validation cohort.

**Conclusion:**

An online nomogram for LSCC was constructed with good predictive performance, which can be used as a practical approach for the personalized early screening and auxiliary diagnosis of the potential risk factors and assist physicians in making a personalized diagnosis and treatment for patients.

## Introduction

Laryngeal squamous cell carcinoma (LSCC) is one of the most common type of head and neck squamous cell carcinoma. In 2020, 12,370 newly discovered Laryngeal cancer cases and 3,750 deaths due to Laryngeal cancer were reported in the United States (1). LSCC originates from the epithelial cells, and the structural and cytological alterations in laryngeal squamous epithelial cells lead to the occurrence of LSCC. Various factors affect the incidence of LSCC; however, the underlying mechanisms remain unclear (2). Treatments of LSCC include surgery, radiotherapy, and chemotherapy. Although the therapeutic modalities have gradually developed over the past two decades, due to the low percentage of early diagnosis, the clinical prognosis has not significantly improved (1). Several patients with LSCC have unremarkable early symptoms, and most of them are only admitted in the hospital if they experience hoarseness and pain during swallowing, progressive aggravation of dysphagia, and radiating ear pain (3). In China, although the popularity of laryngoscopy has increased the rate of early diagnosis of LSCC, laryngoscopy is an invasive procedure, and the incidence of LSCC is relatively low; therefore, laryngoscopy is not used as a routine screening examination for diseases of the pharynx and larynx in the population. However, most of the people living in remote areas in China have poor awareness of the different medical treatments, and the level of medical treatment in these areas is still underdeveloped. Results of previous studies on laryngoscopy performed in this patient group lacked clarity, and white-light endoscopy had a limited ability to detect lesions, which precluded the establishment of an accurate diagnosis (4). The relatively high cost of laryngoscopy led to the underdiagnosis and prevented the early treatment of malignant diseases in the laryngopharynx. In this era of personalized cancer therapy, nomograms are statistical tools that can consider various factors simultaneously to help patients visualize their probability of developing a disease. In addition, nomograms have been several advantages in the treatment of cancer, including personalized assessment, user friendliness, and ease of understanding. However, to our knowledge, no study has developed a dynamic prediction model for LSCC. Therefore, this study aimed to develop an online dynamic nomogram to assist physicians in providing a personalized early diagnosis and treatment of patients with LSCC.

## Materials and Methods

### Patient Data

This study was approved by the Ethics Committee of the First Affiliated Hospital of the Anhui Medical University. All participants provided informed consent. The clinical data of patients with laryngeal diseases admitted in the First Affiliated Hospital of Anhui Medical University, a high-volume surgical center, from April 2017 to October 2020 were obtained. The diagnosis was made based on the results of the postoperative specimen examination performed by two experienced pathologists. Patients (1) with benign laryngeal lesion or stage I to IV LSCC as confirmed by postoperative pathology (2), with complete clinicopathological data, and (3) who signed an informed consent to collect the medical data were included in the study. By contrast, patients (1) who had undergone surgery, chemotherapy, and radiotherapy prior to admission and (2) whose disease was complicated by other malignant tumors, hematologic diseases, active inflammatory diseases (e.g., autoimmune disease and infection), liver and kidney diseases, or long-term use of oral anticoagulant drugs and corticosteroids were excluded. All patients underwent routine physical examination, fibrolaryngoscopy, electrocardiography, and laboratory examination for a comprehensive evaluation. In addition, the clinical data of patients with laryngeal diseases admitted in another high-volume surgical center, Fuyang Hospital, affiliated with Anhui Medical University, were collected.

### Data Collection

The following clinicopathological data were obtained: age, sex, smoking history, and alcohol consumption history. Considering the small volume and less vascular and nerve invasion in most laryngeal diseases, it is difficult to accurately measure them using preoperative computed tomography (CT) and endoscopy; therefore, these relevant imaging findings were not included in this study. Fasting venous blood was collected from patients with laryngeal diseases in the morning within 24 h after admission and was used for routine blood routine and blood biochemical analyses.

### Statistical Analysis

All computations were performed using the R software (version 4.1.2) and various packages. The dataset collected from the First Affiliated Hospital of Anhui Medical University was randomly divided into training and validation cohorts at a ratio of 7:3, and the variables were compared. Non-normal data were presented as median (interquartile ranges). In the univariate analysis, the chi-square test was used to analyze the categorical variables, while the Student’s t-test or rank-sum test was used to examine the continuous variables. In the training cohort, the least absolute shrinkage and selection operator (LASSO) logistic regression analysis was used for multivariate analysis to screen the independent risk factors and build a prediction nomogram for the diagnosis of LSCC (5). The performance of the nomogram was assessed using the receiver operating characteristic (ROC) curve and calibration curve, with the area under the ROC curve (AUC) ranging from 0.5 (no discriminant) to 1 (complete discriminant) (6). A decision curve analysis (DCA) was also performed to determine the net benefit threshold of prediction (7). Spearman’s correlation analysis was performed to analyze the correlations among variables. To facilitate their incorporation into the clinical practice, an interactive web-based dynamic nomogram application was built using Shiny, version 0.13.2.26. Results with a p-value of <0.05 were considered significant.

## Results

### Patient Cohorts and Clinicopathologic Features

**Figure 1:**
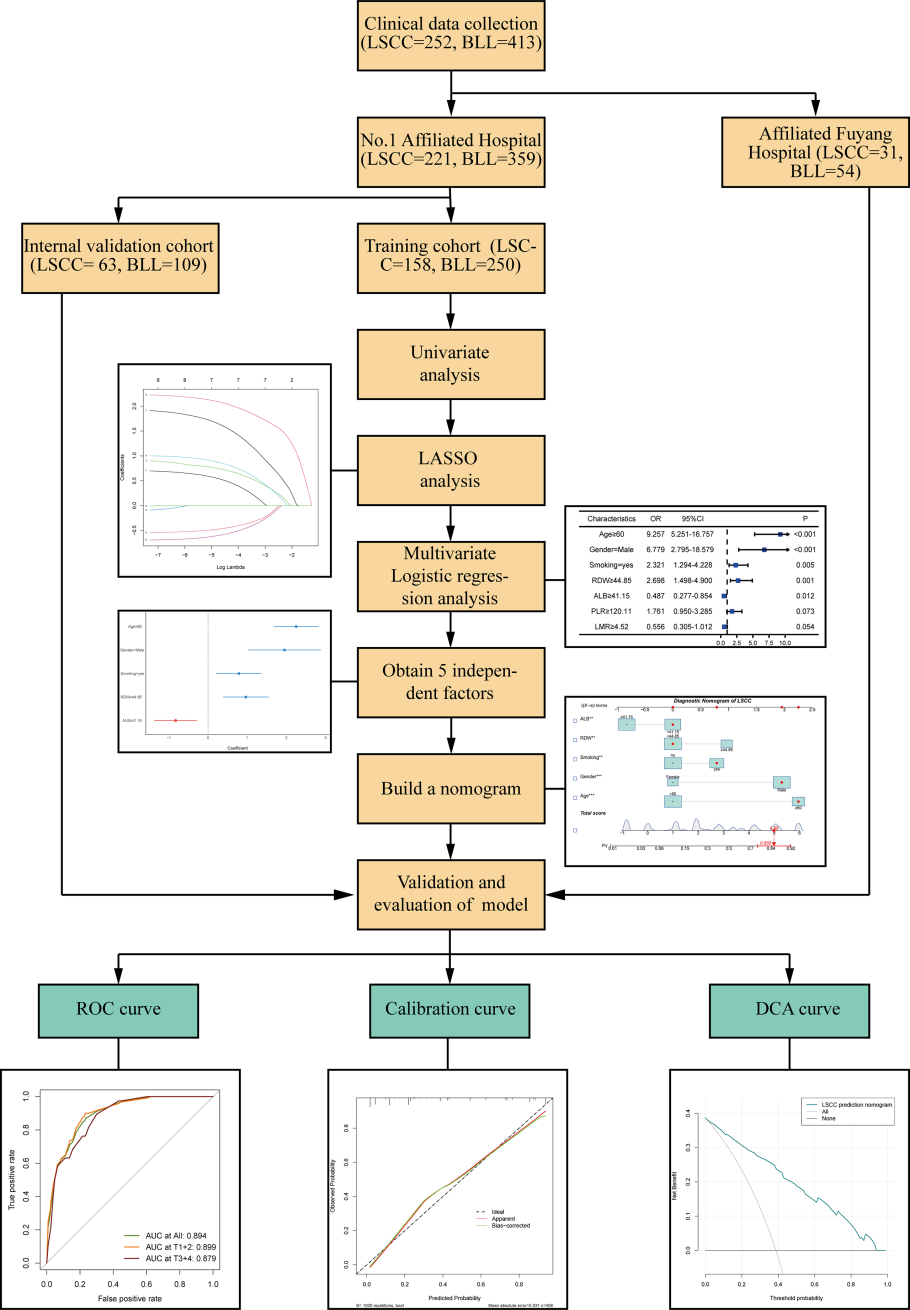
Flow chart of the study process. ** p<0.01, *** p<0.001.

The detailed flow diagram is presented in **Figure 1**. A total of 221 patients with LSCC and 359 patients with benign laryngeal lesions (BLLs), who diagnoses were pathologically confirmed after surgical treatment at the Department of Otolaryngology, Head and neck Surgery of the First Affiliated Hospital of Anhui Medical University from April 2017 to October 2020, and 31 patients with LSCC and 54 patients with BLL who were admitted in Fuyang Hospital affiliated with Anhui Medical University were enrolled in the study. All patients met the inclusion and exclusion criteria. Finally, among the patients included in the study at the First Affiliated Hospital of Anhui Medical University, 70% were selected as the training cohort, while 30% were selected as the internal validation cohort using a computer random method. Patients from Fuyang Hospital affiliated with Anhui Medical University were included in the external validation cohort. The clinicopathological characteristics of the patients are summarized in **Table 1**.

**Table 1:** Patient demographics and clinicopathological characteristics.

Characteristics		The First Affiliated Hospital				Affiliated Fuyang Hospital	
		Training Cohort		Internal Test Cohort		External Test Cohort	
		BLL n (%)	LSCC n (%)	BLL n (%)	LSCC n (%)	BLL n (%)	LSCC n (%)
**All**		250	158	109	63	54	31
**Age (years)**	<60	216 (86.4)	46 (29.1)	100 (91.7)	26 (41.3)	51 (94.4)	12 (38.7)
	≥60	34 (13.6)	112 (70.9)	9 (8.3)	37 (58.7)	3 (5.6)	19 (61.3)
**Gender**	Male	144 (57.6)	151 (95.6)	61 (56.0)	59 (93.7)	23(42.6)	2 (6.5)
	Female	106 (42.4)	7 (4.4)	48 (44.0)	4 (6.3)	31 (57.4)	29(93.5)
**Smoking history**	No	182 (72.8)	59(37.3)	77 (70.6)	20 (31.7)	42 (77.8)	5 (16.1)
	Yes	68 (27.2)	99 (62.7)	32 (29.4)	43 (68.3)	12 (22.2)	26 (83.9)
**Drinking history**	No	201 (80.4)	87 (55.1)	91 (83.5)	34 (54.0)	46 (85.2)	12 (38.7)
	Yes	49 (19.6)	71 (44.9)	18 (16.5)	29 (46.0)	8 (14.8)	19 (61.3)
**Tumor site**	Supra-glottic	50 (20.0)	40 (25.3)	19 (17.4)	20 (31.7)	4 (7.4)	5 (16.1)
	Glottic	200 (80.0)	109 (69.0)	90 (82.6)	41(65.1)	50 (92.6)	26 (83.9)
	Sub-glottic	0 (0)	9 (5.7)	0 (0)	2 (3.2)	0 (0)	0 (0)
**Tumor size**	≤ 2	243 (97.2)	84 (53.2)	100 (91.7)	31(49.2)	54 (100.0)	28 (90.3)
	>2	7 (2.8)	74 (46.8)	9 (8.3)	32 (50.8)	0(0)	3 (9.7)
**T stage**	T1+T2	–	120 (75.9)	–	51 (81.0)	–	22 (71.0)
	T3+T4	–	38 (24.1)	–	12 (19.0)	–	9 (29.0)
**Lymph node metastasis**	N0	–	109 (69.0)	–	51 (81.0)	–	29 (93.4)
	N1	–	18(11.4)	–	4 (6.3)	–	1 (3.2)
	N2	–	29 (18.4)	–	8 (12.7)	–	1 (3.2)
	N3	–	2 (1.3)	–	0 (0)	–	0 (0)
**Distant metastasis**	No	–	155 (98.1)	–	63 (100.0)	–	31(100.0)
	Yes	–	3 (1.9)	–	0 (0)	–	0 (0)
**TNM stage**	I+II	–	95 (60.1)	–	43 (68.3)	–	22 (71.0)
	III+IV	–	63 (39.9)	–	20 (31.7)	–	9 (29.0)
**Differentiation grade**	Well	–	50 (31.6)	–	19 (30.2)	–	26 (83.9)
	Poor	–	33 (20.9)	–	13 (20.6)	–	2 (6.5)
	Moderate	–	75 (47.5)	–	31 (49.2)	–	3 (9.7)

BLL, benign laryngeal lesion; LSCC, laryngeal squamous cell carcinoma.

The Wilcoxon test and chi-square test were used to compare the indices between the LSCC and BLL groups. In the training cohort, nine significant indicators (P<0.05, **Table 2**) were selected, including sex (P<0.001), age (P<0.001), smoking history (P<0.001), drinking history (P<0.001), red blood cell width distribution (RDW, P<0.001), albumin (ALB, P<0.001), neutrophil/lymphocyte ratio (NLR, P<0.001), lymphocyte/monocyte ratio (LMR, P<0.001), and platelet/lymphocyte ratio (PLR, P=0.048).

**Table 2:** Comparison of variables between LSCC group and BLL group.

Variables	The First Affiliated Hospital						Affiliated Fuyang Hospital		
	Training Cohort			Internal Test Cohort			External Test Cohort		
	BLL	LSCC	P	BLL	LSCC	P	BLL	LSCC	P
N	250	158		109	63		54	31	
Gender n (%)			<0.001			<0.001			<0.001
Female	106 (42.4%)	7 (4.4%)		48(44.0%)	4 (6.3%)		32 (59.3%)	2 (6.5%)	
Male	144 (57.6%)	151 (95.6%)		61(56.0%)	59 (93.7%)		22 (40.7%)	29 (93.5%)	
Age n (%)			<0.001			<0.001			<0.001
< 60	216 (86.4%)	46 (29.1%)		100(91.7%)	26 (41.3%)		51(94.4%)	11 (35.5%)	
≥60	34 (13.6%)	112 (70.9%)		9(8.3%)	37 (58.7%)		3 (5.6%)	20 (64.5%)	
Smoking n (%)			<0.001			<0.001			<0.001
No	182 (72.8%)	59 (37.3%)		77(70.6%)	20 (31.7%)		42 (77.8%)	5 (16.1%)	
Yes	68 (27.2%)	99 (62.7%)		32(29.4%)	43 (68.3%)		12 (22.2%)	26 (83.9%)	
Drinking n (%)			<0.001			<0.001			<0.001
No	201 (80.4%)	87 (55.1%)		91(83.5%)	34 (5.0%)		46 (85.2%)	12 (38.7%)	
Yes	49 (19.6%)	71 (44.9%)		18 (16.5%)	29 (46.0%)		8 (14.8%)	19 (61.3%)	
RDW fL (median (IQR))	42.30 (3.80)	44.90 (4.18)	<0.001	42.00 (4.10)	44.30 (4.80)	<0.001	41.80 (3.38)	44.70 (3.95)	0.001
PDW fL (median (IQR))	13.05 (3.78)	13.10 (3.45)	0.551	13.7 (4.00)	13.1 (2.85)	0.131	16.30 (0.50)	16.30 (0.45)	0.616
MPV fL (median (IQR))	10.90 (1.75)	11.10 (1.50)	0.301	11.1 (1.80)	10.9 (1.50)	0.256	11.00 (1.88)	10.10 (2.35)	0.074
PA mg/L (median (IQR))	251.50 (70.00)	239.00 (84.75)	0.062	244.0 (98.00)	264.0 (77.50)	0.094	243.50 (106.25)	224.00 (62.50)	0.169
ALB g/L (median (IQR))	42.10 (4.08)	39.90 (4.73)	<0.001	41.80 (4.30)	41.70 (5.15)	0.346	42.35 (2.65)	41.00 (3.55)	0.001
NLR (median (IQR))	1.87 (0.99)	2.20 (1.14)	<0.001	1.92 (1.05)	2.19 (1.13)	0.029	1.84 (0.62)	2.22 (1.45)	0.043
LMR (median (IQR))	5.22 (2.01)	4.25 (2.23)	<0.001	5.25 (2.40)	4.13 (2.25)	0.001	4.66 (1.53)	3.82 (2.31)	0.258
PLR (median (IQR))	105.48 (46.86)	117.59 (62.06)	0.048	124.56 (56.72)	124.55 (69.57)	0.008	110.97 (40.81)	118.07 (70.22)	0.018

RDW, red blood cell width distribution; PDW, platelet distribution width; MPV, mean platelet volume; PA, prealbumin; ALB, albumin; NLR, neutrophil/lymphocyte ratio; LMR, lymphocyte/monocyte ratio; PLR, platelet/lymphocyte ratio; LSCC, laryngeal squamous cell carcinoma; BLL, benign laryngeal lesions. Red font text means statistically significant.

### Factor Selection for the Predictive Model, Calibration, and Validation of the Nomogram

**Figure 2:**
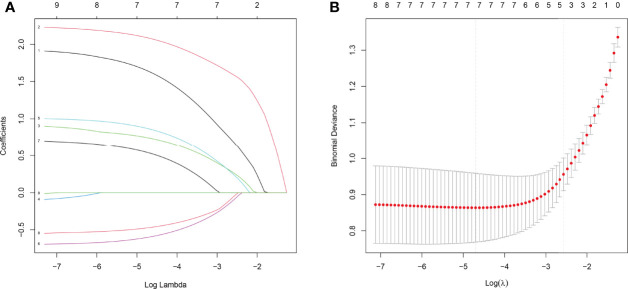
Results of the LASSO regression analysis. **(A)** Plot of the LASSO coefficient profiles. **(B)** Tuning parameter (λ) selection cross-validation error curve.

The above nine variables were included in the original model, which were then reduced to seven potential predictors using LASSO regression analysis performed in the training cohort. The coefficients are shown in **Supplementary Table S1**, and a coefficient profile is plotted in **Figure 2A**. A cross-validated error plot of the LASSO regression model is shown in **Figure 2B**. As shown in **Figure 2B**, the most regularized and parsimonious model, with a cross-validated error within one standard error of the minimum, included seven variables.

**Figure 3:**
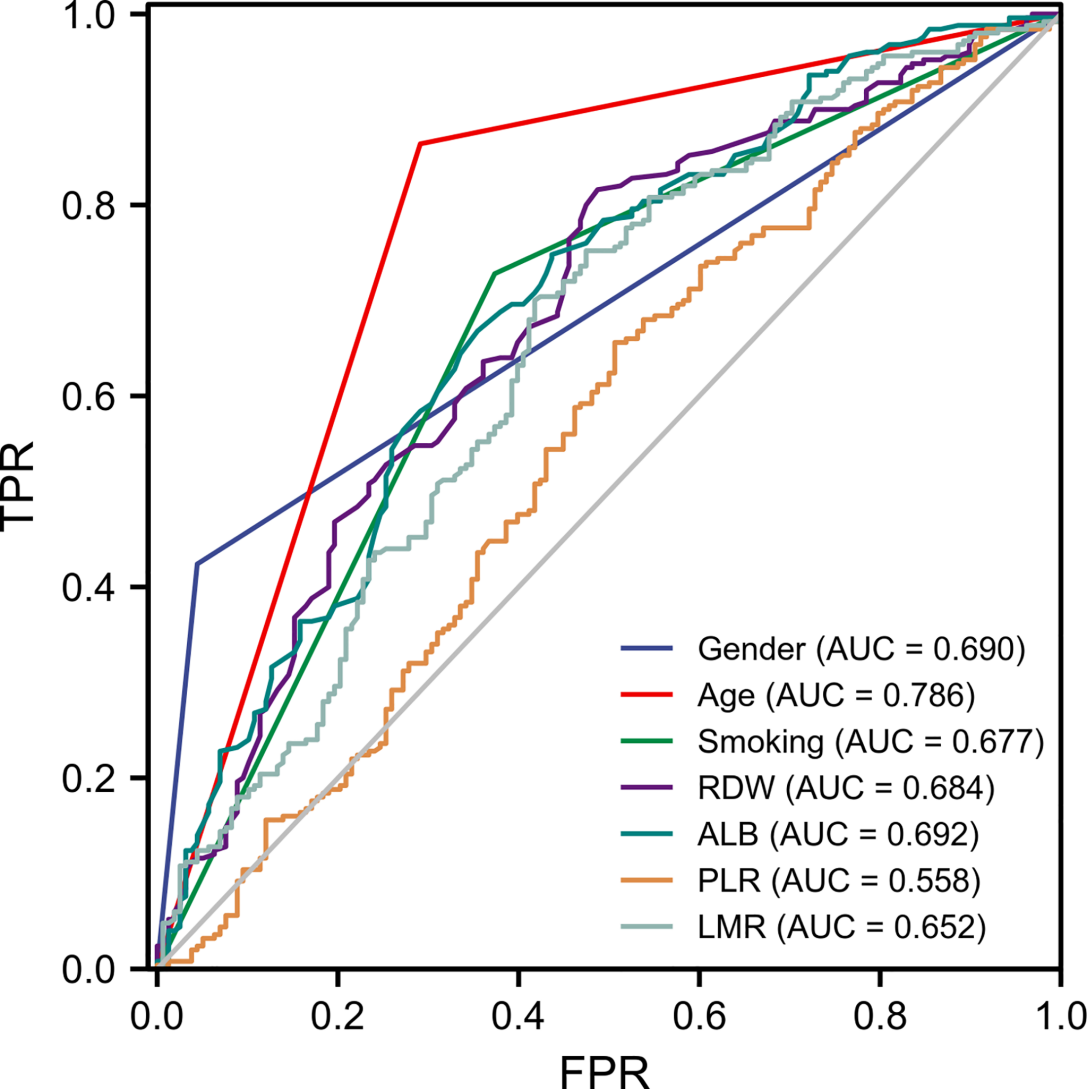
ROC curve analysis of seven candidate diagnostic indicators.

As shown in **Figure 3**, the ROC analysis of the abovementioned variables yielded AUC values greater than 0.5 for sex (AUC=0.690), age (AUC=0.786), smoking (AUC=0.677), RDW (AUC=0.684), ALB (AUC=0.692), LMR (AUC=0.652), and PLR (AUC=0.558). The cutoff values, sensitivity, and specificity of these parameters are shown in **Table 3**.

**Table 3:** The Results of receiver operating characteristic (ROC) curve.

Variables	AUC	Cut-off value	Youden index	Sensitivity	Specificity	95%CI	P-Value
Gender	0.690	–	0.380	0.424	0.956	0.655-0.725	<0.001
Age	0.786	–	0.573	0.864	0.709	0.745-0.828	<0.001
Smoking	0.677	–	0.355	0.728	0.627	0.630-0.724	<0.001
RDW	0.684	44.85	0.329	0.816	0.513	0.631-0.738	<0.001
ALB	0.692	41.15	0.314	0.668	0.646	0.639-0.745	<0.001
LMR	0.652	4.52	0.282	0.700	0.582	0.597-0.708	<0.001
PLR	0.558	120.11	0.150	0.656	0.494	0.499-0.617	0.048

RD, red blood cell width distribution; ALB, albumin; LMR, lymphocyte/monocyte ratio; PLR, platelet/lymphocyte ratio.

**Figure 4:**
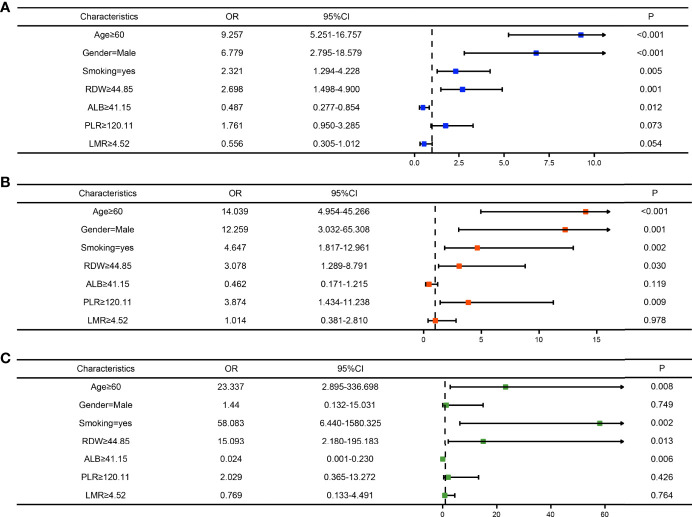
Forest maps of the logistic regression analysis of the training cohort **(A)**, internal test cohort **(B)**, and, external test cohort **(C)**.

**Figure 5:**
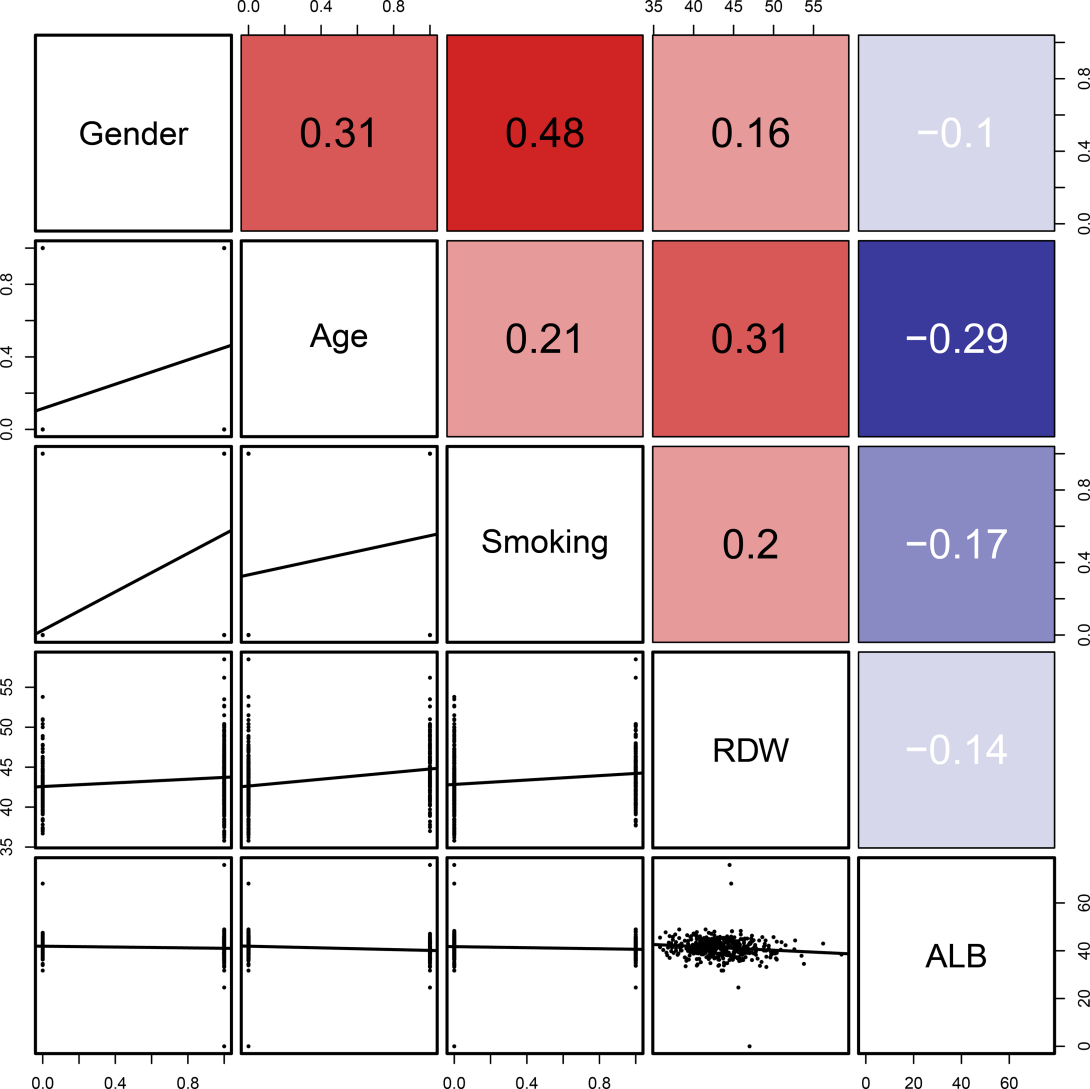
Linear correlation analysis of the five indicators (age, sex, smoking history, RDW, and ALB). The number in the right of the plot was the correlation coefficient.

To clarify whether the abovementioned seven variables were independent risk factors for LSCC, further multivariate logistic analysis excluding other confounding factors was carried out and showed that age (odds ratio [OR]=9.257, 95% confidence interval [CI]=5.251–16.757, P<0.001; **Figure 4A**), sex (OR=6.779, 95% CI=2.795–18.579, P<0.001; **Figure 4A**), smoking history (OR=2.321, 95% CI=1.294–4.228, P=0.005; **Figure 4A**), RDW (OR=2.698, 95% CI=1.498–4.900, P=0.001; **Figure 4A**), and ALB (OR=0.487, 95% CI=0.277–0.854, P=0.012; **Figure 4A**) were significantly associated with LSCC. Similar results were obtained in the internal and external validation cohorts (**Figures 4B** **, C**). Results of the correlation analysis showed that the five factors were all linearly correlated with each other, while ALB was negatively correlated with other indices (**Figure 5**).

**Figure 6:**
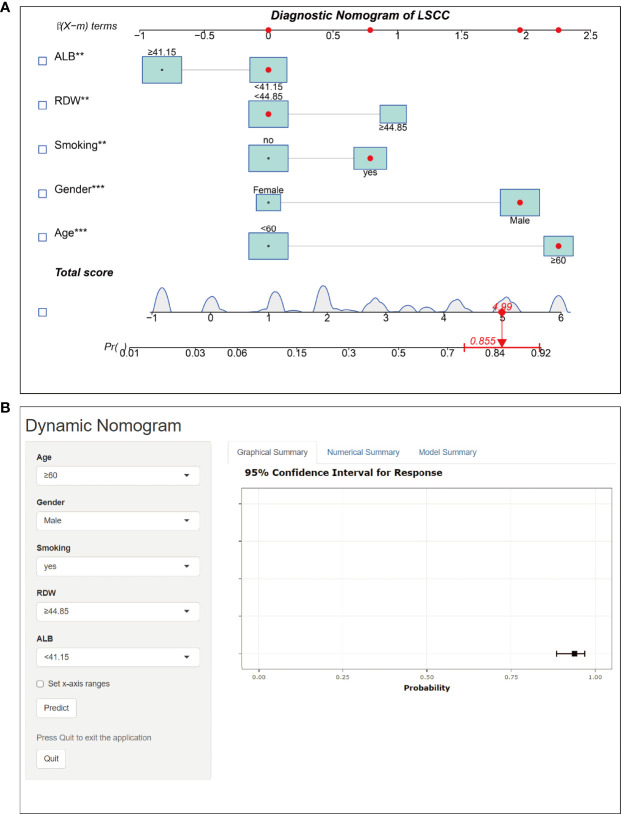
Nomogram prediction model for LSCC diagnosis. **(A)** Established nomogram in the training cohort by incorporating the following five parameters: age, sex, smoking history, RDW, and, ALB.** p<0.01, *** p<0.001. **(B)** Online dynamic nomogram accessible at https://hanchenchen.shinyapps.io/LSCCNomapp/.

The final logistic model included five independent predictors (age, sex, smoking history, RDW, and ALB) and was developed as a simple-to-use nomogram, which is illustrated in **Figure 6A** and available online (https://hanchenchen.shinyapps.io/LSCCNomapp/) and presented in **Figure 6B**. The specific coefficients of each factor are shown in **Supplementary Figure S1**.

**Figure 7:**
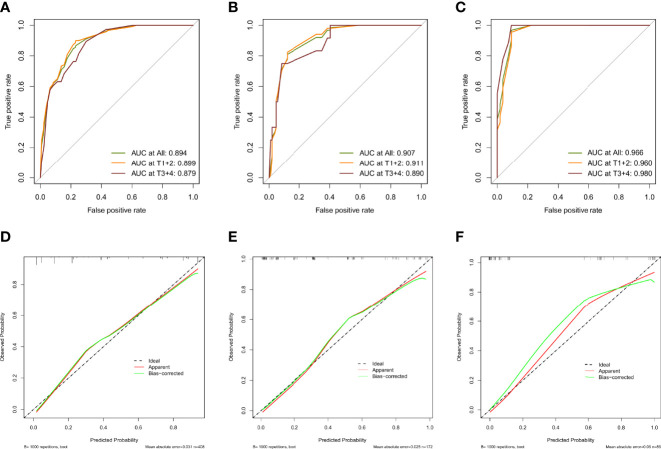
Evaluation of validity and reliability of the model. ROC curves of the nomogram prediction model in the training cohort **(A)**, internal test cohort **(B)**, and, external test cohort **(C)**; calibration curves of the nomogram prediction model for the training cohort **(D)**, internal test cohort **(E)**, and, external test cohort **(F)**.

As shown in **Figures 7A–C**, the AUCs of the model in the training cohort, internal validation cohort, and external validation cohort were 0.894, 0.907, and 0.966, respectively, showing good predictive ability. In addition, we also calculated the model AUC of early T stage (T1+T2) patients in three cohorts specifically, which were 0.899, 0.911 and 0.960, respectively, suggesting that nomogram may play an important role in the early screening of LSCC. The internal validation and calibration of the nomogram were performed using 1,000 bootstrap analyses. The calibration plots of the nomogram in the three cohorts are plotted in **Figures 7D–F**, which demonstrate a good correlation between the observed and predicted development of LSCC. The above results showed that the original nomogram was still valid for use in the inner and outer validation sets, and the calibration curve of this model was relatively close to the ideal curve, which indicates that the predicted results were consistent with the actual findings.

### Decision Curve Analysis

**Figure 8:**
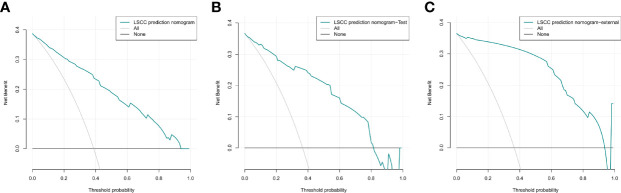
Decision curve analysis of the nomogram of the training cohort **(A)**, internal test cohort **(B)**, and, external test cohort **(C)**.

The DCA curves for the nomogram are presented in **Figures 8A** **–C**. A high-risk threshold probability is the probability of serious deviation in the prediction of the model when clinicians have serious defects using a nomogram for diagnosis and decision-making. In this study, the DCA curve demonstrated that the nomogram had good net benefits for clinical use.

## Discussion

More than 80,000 people die of laryngeal cancer every year worldwide, and LSCC accounts for approximately 90% of all laryngeal malignancies. Although the therapeutic modalities for LSCC, such as radiotherapy, chemotherapy, and surgical techniques, have made tremendous progress, the survival rate of patients with LSCC remains stagnant over the past 30 years due to the low rate of early diagnosis (8). In 2020, the European Society of Oncology suggested that the diagnosis of LSCC should not only be based on the patient’s medical history, complete physical examination results, electronic fiber laryngoscopy findings, enhanced CT or magnetic resonance imaging findings, and pathological diagnosis but should also include the evaluation of biochemical and hematological indicators (9). However, for some patients with laryngeal cancer, especially those with early-stage laryngeal cancer (TNM stage I + II), most have limited and small lesions, and preoperative CT and endoscopy cannot accurately determine their size, morphology, local invasion, and the safe margin of the lesions. Many lesions develop in the deep areas of the mucosa and show endophytic expansive growth. The mucosal surface is smooth and regular. The patient’s irritated choking reflex was very strong on preoperative biopsy, which reduced its success rate and accuracy. Therefore, for patients with difficulty in differentiating laryngeal cancer from benign lesions of the larynx (vocal fold polyps, vocal Reinke’s edema, laryngeal knot nuclei, papilloma of the larynx, keratosis of the larynx, amyloidosis of the larynx, etc.), the establishment of a nomogram for preoperative prospective quantitative prediction can help overcome these problems and allow the surgeon to formulate an individualized surgical approach for patients preoperatively. In this study, age, sex, smoking history, preoperative nutritional status (ALB), and preoperative RDW were significant predictors, and these independent factors influenced the occurrence and development of laryngeal diseases; moreover, the calibration plot of the nomogram closely matched the ideal standard line, indicating that the nomogram had sufficient statistical power to predict the incidence of diseases. Due to the inconvenience of traditional nomograms for clinical use, online versions of the nomograms were built based on traditional nomogram models. Online versions can be easily accessed by computers, smartphones, or other mobile devices and more effectively provide accurate and individualized diagnosis prediction for patients with LSCC. Therefore, in the diagnosis and treatment of malignant and benign diseases of the larynx, this model provides a practical and convenient navigation tool for preoperative propensity diagnosis, individualized treatment implementation, surgical modality selection, and clinical trial design.

In our study, age ≥60 years was an independent risk predictor for the development of LSCC, which was consistent with the result of most previous studies (10). Relevant studies have shown that the age-standardized incidence rate of laryngeal cancer by the world standard population (ASIRW) is 2.0/100,000. However, the ASIRW was 3.6/100,000 for men and 0.48/100,000 for women. This notion was also confirmed by our study that malignant laryngeal lesions were more common in men than in women; therefore, sex was also included in the nomogram (11). Although female patients with malignant laryngeal lesions are generally older, most are in the postmenopausal state, while a small proportion are in the premenopausal state. Age is associated with menopausal status, indicating that it might complicate the relationship between menopausal status and the development of laryngeal cancer (12). However, due to the small sample of female patients with laryngeal cancer, information on reproduction, hormone levels, use of oral contraceptives, and menopausal hormone replacement therapy status in female patients was not taken into account. Moreover, a correlation analysis was not performed in this study; hence, further exploration of the action of estrogen and its receptor could provide new insights into the diagnosis and treatment of female patients with laryngeal cancer.

Laryngeal cancer belongs to the group of tobacco-dependent malignancies including passive smoking. This cancer rarely occurs among nonsmokers (13). The increased risk of laryngeal cancer among individuals who started smoking at an earlier age is mainly due to the longer duration of smoking and higher cumulative tobacco exposure (14). Smoking cessation reduces the probability of developing laryngeal cancer, especially among former smokers who have quit smoking for 15 or more years (15). Tobacco increases the relative risk of developing laryngeal cancer in women than in men (12). This study showed that a history of smoking was a significant risk factor for the development of laryngeal cancer, and the possible mechanisms were determined. Many chemicals in tobacco have toxic effects, including polycyclic aromatic hydrocarbons (phenyltoluene), N-nitrosamines, heavy metals (nickel, cadmium, chromium, and arsenic), alkaloids (nicotine and its main metabolites, and infectious agents), and aromatic amines. Tobacco induced LSCC pathogenesis including inflammatory and immune changes, genetic alterations, oxidative damage, endothelial dysfunction, and cellular senescence (16). A significant difference was observed in the rate of drinking (45.2%, 71/221) and non-drinking (67.9%, 150/221) among the patients with LSCC admitted to the First Affiliated Hospital of Anhui Medical University. However, history of drinking was not an independent predictor of laryngeal cancer in our study, although most studies suggest that long-term heavy drinking is an independent risk factor for the development of laryngeal cancer (17), which is inconsistent with the findings of our study. Due to the insufficient sample size and regional dietary cultural differences, there may be deviations in the results of this study, which can be further evaluated by extending the coverage of the center and expanding the sample size in the future.

Chronic inflammation and lack of nutrition are closely related to the occurrence and development of various malignant tumors. Chronic inflammation induces tumor angiogenesis and DNA damage, and promotes tumor proliferation and metastasis by preventing apoptosis (18). However, the diagnosis of LSCC based on the changes in inflammatory biomarkers in the peripheral blood has not yet been reported. In patients with LSCC, the ALB levels were significantly reduced, while the RDW levels were significantly increased, both of which were independent risk factors for LSCC; this finding suggests that ALB and RDW are potential biomarkers for the diagnosis of LSCC.

Previous clinical applications of RDW were limited to the diagnosis of iron-deficient anemia. Recent studies have revealed elevated RDW levels in patients with cardiovascular diseases, venous thromboembolism, rheumatoid arthritis, diabetes, and cancer. RDW was positively associated with the levels of plasma inflammatory biomarkers (C-reactive protein (19, 20), erythrocyte sedimentation rate (21), and interleukins), which are considered inflammatory tumor biomarkers. Elevated RDW levels were probably related to the release of inflammatory factors (e.g., IL-6 and IFN-γ) that could inhibit EPO production, resulting in an increased proportion of immature erythroblasts in the peripheral blood (22, 23). In addition, elevated RDW levels are biomarkers of inflammation and oxidative stress-induced damage, which affects the occurrence and development of various types of cancer by maintaining proliferation signals, escaping growth inhibitors, resisting cell death, inducing angiogenesis, and activating invasion and metastasis (24, 25). The lack of nourishment, including various mineral and vitamin deficiencies (e.g., iron, folic acid, and vitamin B12) in patients with cancer, increases the RDW levels (26). Recent studies have found that RDW levels in patients with colon cancer are significantly higher than those in patients with colon polyps (27–29). Moreover, patients who developed esophageal cancer, breast cancer, lung cancer, gastric cancer, colon cancer, prostate cancer, lymphoma, and other malignant tumors with high RDW levels have a poor prognosis (28, 30, 31). Hence, RDW levels indicate chronic inflammation and malnutrition in patients with cancer. However, there is a lack of relevant research evaluating the role of RDW levels in the diagnosis, staging, and metastasis of LSCC. In this study, ROC curve analysis showed that the AUC of RDW in the diagnosis of LSCC was 0.684, suggesting a diagnostic significance for LSCC.

As a water-soluble liver protein, ALB acts as a transporter of several hormones, minerals, and fatty acids, and helps maintain the capillary colloidal osmotic pressure. ALB acts as an antioxidant in the plasma and interstitial space, providing amino acids for matrix deposition and cell proliferation (32). ALB is the primary protective factor against stable DNA replication and cell growth. High concentrations of ALB significantly inhibits the growth of various tumor cells. The inflammatory response and nutrition in patients with cancer can lead to a reduction in ALB production (33, 34). For instance, cancer promotes the expression of TNF-α (a pro-inflammatory factor), which can downregulate the transcription of the albumin gene and inhibit albumin synthesis in hepatocytes. However, TNF-α also increases the permeability of microvessels and exudation of albumin through the capillaries (35, 36), reducing the ALB levels. Previous clinical studies have shown that cancer patients usually present with a severe nutritional status at the time of diagnosis. Innutrition is known to play a vital role in the occurrence and development of cancer cachexia as well as the recurrence and progression of various cancer types (37). Hence, low levels of ALB in patients with different malignant tumors have been associated with poor prognosis (34, 38), which was consistent with our results. In this study, the ALB levels in the LSCC group were significantly lower than those in the BLL control group. Additionally, the cut-off value of ALB in the diagnosis of LSCC was 41.15, with a sensitivity of 66.8%, a specificity of 64.6%, and a 95% CI of 0.639–0.745, thus indicating that it was an excellent diagnostic indicator.

Taken together, we conclude that ALB and RDW are potential biomarkers for the auxiliary diagnosis of LSCC. Independent analysis showed that both inflammation-related indicators were independent predictors of risk factors for LSCC. Therefore, by combining these two inflammatory biomarkers with other clinical factors (age, sex, and smoking history), an online predictive nomogram model was constructed, which is valuable in the auxiliary diagnosis and may be useful for early treatment of LSCC.

However, our study some limitations. First, this was a retrospective study with some inevitable bias. Therefore, a multicenter randomized controlled clinical study with a larger sample size could be performed in the future to verify the clinical benefits. Moreover, whether the incidence of laryngeal cancer in women is correlated with menopausal status and estrogen levels should be explored further. Second, the prediction model and dynamic nomogram were based on known risk factors, but some factors influencing the incidence of LSCC have not been studied and justified. Hence, relevant indicators can be continuously refined in the future with the development of molecular biology, which can further improve the diagnostic accuracy of the dynamic online nomogram. Third, the establishment of the model is based on the perioperative data; therefore, preoperative prediction is impossible. Finally, this algorithm only considered patients undergoing surgery; therefore, there may be selection bias compared with other nomograms.

## Conclusion

This study was the first to develop and validate online nomograms based on the independent risk factors to dynamically predict diagnosis in individuals with LSCC. These novel models demonstrated superior performance and discriminative power, which can provide vital information for otolaryngologists when designing customized clinical treatments.

## Data Availability Statement

The original contributions presented in the study are included in the article/supplementary material. Further inquiries can be directed to the corresponding authors.

## Ethics Statement

The studies involving human participants were reviewed and approved by the medical ethics committees of The First Affiliated Hospital of Anhui Medical University (Reference number: Quick -PJ 2021-15-32). The patients/participants provided their written informed consent to participate in this study.

## Author Contributions

All the authors worked together to complete the paper. YeL, as the Corresponding Authors guided the design of the entire experiment and the writing of the paper. Yu L, YH, and BC are responsible for the collection of specimens, the writing of papers, design of the experiment, and other scattered work, they contributed equally to this work as first authors. YT, JW, DL, ZD, ZF, SY, JPW, KY, JZ, HS, YW, XW, and YJ were engaged in the collection of the clinical data. All authors contributed to the article and approved the submitted version.

## Funding

This project was supported by National Natural Science Fund (82171127).

## Conflict of Interest

The authors declare that the research was conducted in the absence of any commercial or financial relationships that could be construed as a potential conflict of interest.

## Publisher’s Note

All claims expressed in this article are solely those of the authors and do not necessarily represent those of their affiliated organizations, or those of the publisher, the editors and the reviewers. Any product that may be evaluated in this article, or claim that may be made by its manufacturer, is not guaranteed or endorsed by the publisher.

## Supplementary Material

Supplementary Figure 1 The coefficients of multivariate logistic regression analysis.

Supplementary Table 1 The coefficients of Lasso regression analysis.

## Abbreviations

AUC, area under the ROC curve; BLL, benign laryngeal lesions; CI, confidence interval; DCA, decision curve analysis; LASSO, least absolute shrinkage and selection operator; LSCC, Laryngeal squamous cell carcinoma; OR, odds ratio; RDW, red blood cell width distribution; ROC, receiver operating characteristic.
