# Supplementary figures and images for: A New Online Dynamic Nomogram: Construction and Validation of an Assistant Decision-Making Model for Laryngeal Squamous Cell Carcinoma
Source: Front Oncol. 2022 May 26;12:829761. doi: 10.3389/fonc.2022.829761 (PMC9204277; doi:10.3389/fonc.2022.829761)

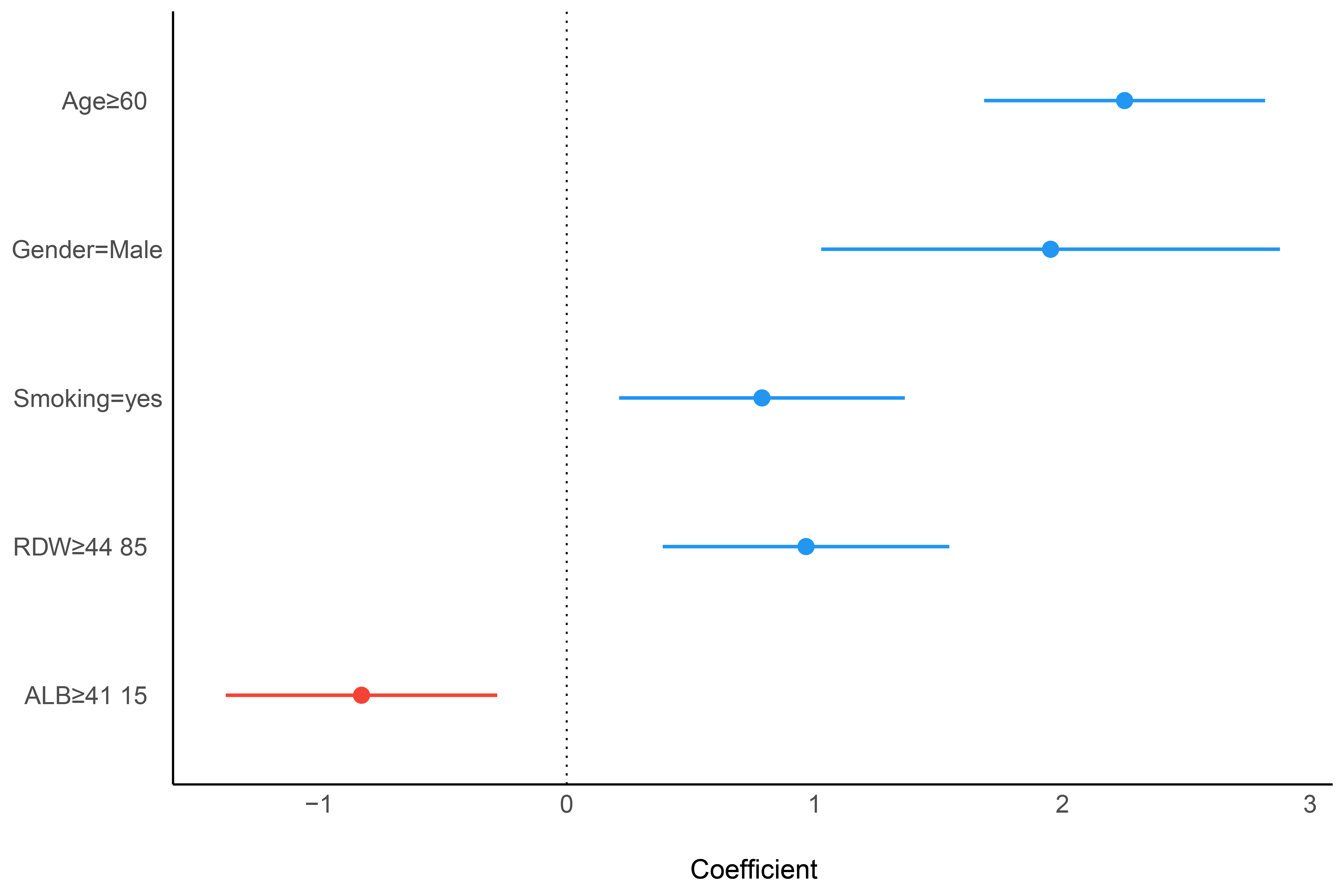

Supplement: Supplementary Figure 1 — The coefficients of multivariate logistic regression analysis. [file Image_1.tif]
