# Supplementary material for: A New Online Dynamic Nomogram: Construction and Validation of an Assistant Decision-Making Model for Laryngeal Squamous Cell Carcinoma
Source: Front Oncol. 2022 May 26;12:829761. doi: 10.3389/fonc.2022.829761 (PMC9204277; doi:10.3389/fonc.2022.829761)
Supplement: Supplementary Table 1 — The coefficients of Lasso regression analysis. [file Table_1.docx]

**Table S1.** The coefficients of Lasso regression analysis.

| **Variables** | **Coefficients** |
| --- | --- |
| Gender | 1.7148250 |
| Age | 2.1249747 |
| Smoking | 0.7752295 |
| Drinking | . |
| RDW | 0.9085750 |
| ALB | -0.6294917 |
| PLR | 0.5842692 |
| LMR | -0.4940403 |
| NLR | . |
